# Effects of Six Months of Levothyroxine Therapy on Sympathovagal Imbalance and Cardiometabolic Profile in Overt Hypothyroid Patients

**DOI:** 10.7759/cureus.81268

**Published:** 2025-03-27

**Authors:** A Naga Syamsundara Kiran, Gopal Krushna Pal, Pravati Pal, Sadishkumar Kamalanathan, Subhash Parija, Mohammed Jaffer Pinjar

**Affiliations:** 1 Physiology, All India Institute of Medical Sciences, Kalyani, Kalyani, IND; 2 Physiology, Jawaharlal Institute of Postgraduate Medical Education and Research, Puducherry, IND; 3 Endocrinology, Jawaharlal Institute of Postgraduate Medical Education and Research, Puducherry, IND; 4 Medical Microbiology, Shri Balaji Vidyapeeth, Puducherry, IND; 5 Physiology, All India Institute of Medical Sciences, Deoghar, IND

**Keywords:** cardiovascular risks, levo-thyroxine therapy, overt hypothyroidism, oxidative stress and inflammation, sympathovagal imbalance

## Abstract

Introduction: Hypothyroidism, a common endocrine disorder, is linked to cardiovascular risks arising from autonomic imbalance and metabolic dysregulation. While overt hypothyroidism (OH) manifests distinct thyroid hormone abnormalities, subclinical hypothyroidism (SCH) presents milder hormonal changes. Levothyroxine therapy is widely used for thyroid function restoration, but its long-term effects on autonomic and cardiovascular health in OH remain understudied. This study investigates the therapeutic effects of six months of levothyroxine treatment on autonomic function and metabolic parameters in OH patients.

Materials and methods: A follow-up study was conducted on OH patients receiving levothyroxine therapy. Participants with confounding cardiovascular comorbidities were excluded. Clinical assessments included autonomic function tests, metabolic profiling (lipid and thyroid parameters), and inflammatory/oxidative stress markers. Comparative analyses were performed against healthy controls.

Results: Levothyroxine therapy effectively restored thyroid hormone levels in OH patients. Autonomic function tests demonstrated improved parasympathetic modulation and partial sympathovagal balance recovery, though residual autonomic irregularities persisted. Lipid profiles showed marked improvement but did not fully normalize compared to controls. Inflammatory and oxidative stress markers decreased significantly post-therapy, yet remained elevated relative to healthy individuals. Statistical modeling identified oxidative stress as a key contributor to autonomic dysfunction.

Discussion: While levothyroxine normalized thyroid function and improved autonomic balance, incomplete resolution of metabolic and inflammatory abnormalities suggests persistent cardiovascular risks in OH patients after six months of therapy. The findings highlight the need for extended treatment durations to achieve comprehensive cardiovascular risk mitigation.

Conclusion: Despite therapeutic benefits, OH patients retain residual cardiovascular risks post-levothyroxine therapy, necessitating long-term monitoring. Future research should investigate optimal treatment durations and adjunct therapies to address persistent autonomic and metabolic dysfunction in this population.

## Introduction

India, one of the most populous countries in the world, faces an increasing burden of non-communicable diseases. Hypothyroidism is a prevalent endocrine disorder globally, with overt hypothyroidism (OH) and subclinical hypothyroidism (SCH) affecting 10.95% and 8.02% of the Indian population, respectively [1]. OH is characterized by elevated thyroid-stimulating hormone (TSH) and reduced free thyroxine (fT4), while SCH involves elevated TSH with normal fT4 levels [2].

Thyroid hormones play a crucial role in increasing basal metabolic rate (BMR) and tissue thermogenesis, influencing almost all tissues and organ systems. BMR is elevated through the upregulation of alpha-adrenergic receptors, which enhances sympathetic activity. Consequently, hyperthyroidism is associated with heightened sympathetic activation, while hypothyroidism typically shows diminished sympathetic responses [3,4]. Paradoxically, hypothyroidism can lead to sympathovagal imbalance (SVI) due to increased sympathetic tone [5,6]. Previous studies have confirmed elevated sympathetic and reduced parasympathetic activity in both OH and SCH patients [7,8], with SVI linked to cardiovascular complications [9]. Additionally, both OH and SCH are associated with cardiovascular disease (CVD) risk factors, such as dyslipidemia [10].

The thyroid gland secretes approximately 80% thyroxine (T4) and 20% triiodothyronine (T3) [11]. In hypothyroidism, this secretion process is disrupted, resulting in decreased fT4 levels. Levothyroxine (L-thyroxine), a synthetic form of T4, is the primary treatment for hypothyroidism. It mimics the endogenous production of T4 by the thyroid gland [12].

While SCH is a mild thyroid dysfunction with normal T4 levels and elevated TSH, OH is characterized by reduced T4 levels, indicating severe thyroid failure [2]. OH presents with more severe symptoms, such as fatigue, cold intolerance, weight gain, dry skin, and an increased risk of CVD [13]. Although L-thyroxine is the preferred treatment for both SCH and OH, research predominantly focuses on SCH, leaving a gap in understanding the long-term effects of L-thyroxine on SVI and cardiovascular complications in OH patients. This study investigates the therapeutic effects of six months of levothyroxine treatment on autonomic function and metabolic parameters in OH patients.

## Materials and methods

This follow-up study was conducted after obtaining approval from the JIPMER Scientific Advisory Committee (JSAC) and the Institutional Ethics Committee for Human Studies at JIPMER, Puducherry. Patients were recruited from the endocrinology outpatient department (OPD) of JIPMER during routine check-ups. The inclusion criteria were patients diagnosed with OH and undergoing treatment with L-thyroxine for a minimum of six months to two years. Patients with conditions affecting cardiovascular functions, such as hypertension, heart diseases, autonomic failure, diabetes mellitus, or other endocrine disorders, as well as those on chronic medications, were excluded. Age- and gender-matched healthy volunteers were recruited as the control group for comparison.

**Figure 1:**
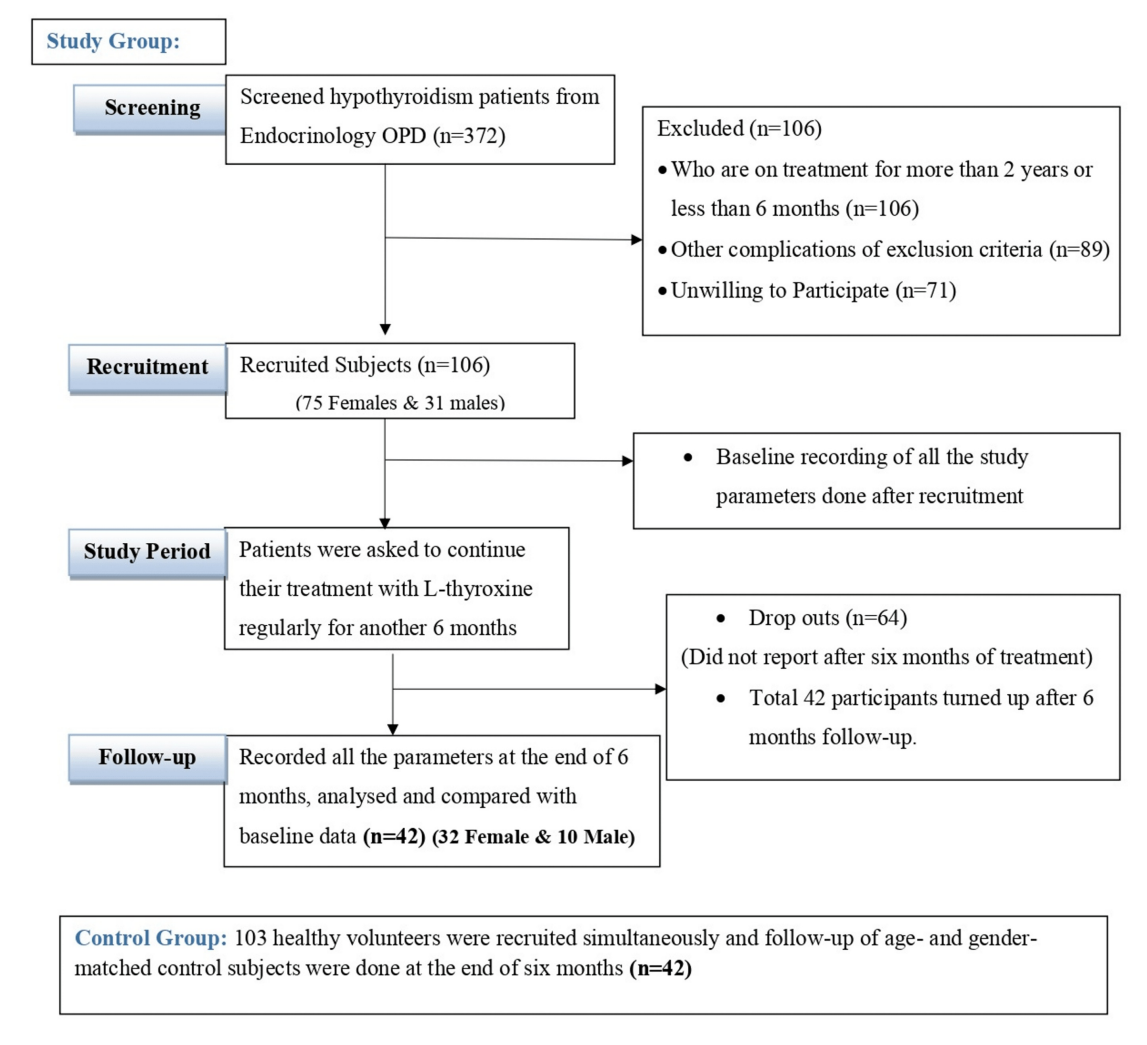
Consort diagram of participant recruitment and follow-up

After obtaining written informed consent, a total of 106 overt hypothyroid patients were enrolled in the study. However, only 42 participants returned for follow-up after six months (Figure 1). 

Recording of study parameters for SVI

Anthropometric parameters and basal blood pressure were recorded with the patient in a supine position. Short-term heart rate variability (HRV) was assessed by recording a 5-10-minute electrocardiogram (ECG) after the patient rested comfortably for 10 minutes. Standard procedures recommended by the Task Force on HRV were followed [14]. SVI and autonomic reactivity were assessed using cardiovascular autonomic function tests (CAFTs) [15,16]. Baroreceptor reflex sensitivity (BRS) and other cardiovascular parameters were recorded using the continuous blood pressure variability (BPV) method with Finapres [17,18].

Recording of biochemical parameters

The fasting blood sample was collected during recruitment, and biochemical parameters like Free-T3, Free-T4, and TSH were estimated. Every morning at 07:30 AM, the fasting sample was collected from each study participant. In addition, lipid profile parameters like total cholesterol (TC), triglycerides (TG), and HDL cholesterol were also analyzed. Atherogenic index (AI) was estimated using the formula log10 (TG/HDL) [19]. The markers, like high-sensitivity C-reactive protein (hs-CRP), anti-thyroperoxidase antibody (TPO) titer, anti-thyroglobulin antibody (Tg) titer, and malondialdehyde (MDA), were estimated by the colorimetric method using ELISA (enzyme-linked immunosorbent assay) kits.

Levothyroxine therapy

At baseline, patients were instructed to continue taking L-thyroxine ("Eltroxin" tablets) for six months. The medication was to be taken on an empty stomach at least 30 minutes before breakfast [20]. Patients were followed up every three months, with serum fT4 and TSH levels measured during these visits. At the end of six months, all cardiovascular and biochemical parameters were reassessed.

Statistical analysis

Data were analyzed using SPSS version 19 (IBM Corp, Armonk, NY). Intergroup differences in means between controls and OH patients were compared using Student's unpaired t-test for normally distributed data, and the Mann-Whitney test was used for non-normally distributed data. Multiple regression analysis was performed to evaluate the independent contributions of metabolic factors, such as AI, BMI, and oxidative stress parameters, to sympathovagal balance (alterations in LF-HF [ratio of low-frequency to high-frequency power] ratio). Data are presented as mean ± SD, and a p-value of less than 0.05 was considered statistically significant.

## Results

There was no significant difference in the basal parameters between before and after six months in the control group. After six months of L-thyroxine therapy, there was no significant difference in the basal heart rate and SBP in the overt hypothyroid group. However, BMI, DBP, and MAP were significantly decreased (P<0.001) in them after six months of L-thyroxine therapy (Table 1).

**Table 1:** Comparison of BMI, heart rate and blood pressure parameters of overt hypothyroid groups between before and after six months of L-thyroxine therapy compared with controls. Data presented are mean±SD. The P values less than 0.05 were considered statistically significant. *Indicates the comparison between before six months and after six months in the overt hypothyroid group (*P<0.05, **P<0.01; ***P<0.001). ^#^Indicates comparison of before six months of the hypothyroid group with before six months of the control group (^#^P<0.05, ^##^P<0.01; ^###^P<0.001). BMI: body mass index; BHR: basal heart rate; SBP: systolic blood pressure; DBP: diastolic blood pressure; MAP: mean arterial pressure.

Parameters	Control group		Overt hypothyroid group	
	Before 6 months (n=42)	After 6 months (n=42)	Before 6 months (n=42)	After 6 months (n=42)
BMI (kg/m)	21.94±3.83	22.42±3.72	27.02±3.61^###^	22.69±4.67***
BHR (per min)	73.955±8.80	74.25±7.58	71.92±4.19	75.14±14.52
SBP (mmHg)	107.25±9.23	106.50±8.46	109.57±3.10	109.83±8.53
DBP (mmHg)	67.5±8.09	68.82±8.20	77.92±7.23^###^	69.19±7.11***
MAP (mmHg)	81.20±7.25	80.34±6.92	88.55±4.87^###^	82.73±6.76***

Autonomic function****

Frequency and time domain indices of HRV did not show any significant difference between the pre-treatment and six-month post-treatment periods in the control group (Tables 2, 3). However, there was a significant increase in total power (TP) and high-frequency power in normalized units (HFnu) (Table 2) and a significant decrease in low-frequency power in normalized units (LFnu) and LF:HF ratio of frequency domain indices (Table 2) between the pre-treatment period and after six months of L-thyroxine therapy in the overt hypothyroid group.

**Table 2:** Comparison of frequency domain indices of heart rate variability parameters of overt hypothyroid groups between the pre-treatment period and after six months of L-thyroxine therapy compared with controls Data presented are mean±SD. The P values less than 0.05 were considered statistically significant. *Indicates comparison between before six months and after six months in the control group and comparison between before six months and after six months in the overt hypothyroid group (*P<0.05, **P<0.01; ***P<0.001). ^#^Indicates comparison of before six months of hypothyroid group with before six months of control group (^#^P<0.05, ^##^P<0.01; ^###^P<0.001). ^†^Indicates comparison of after six months of hypothyroid group with after six months of control group (^†^P<0.05, ^††^P<0.01; ^†††^P<0.001). TP: total power; LFnu: low-frequency power in normalized units; HFnu: high-frequency power in normalized units; LF-HF: ratio of low-frequency to high-frequency power.

Parameters	Control group		Overt hypothyroid group	
	Before 6 months (n=42)	After 6 months (n=42)	Before 6 months (n=42)	After 6 months (n=42)
TP (m s^2^)	801.86±288.0	792.60±252.80	380.04±77.2^###^	509.55±133.2*^,†††^
LFnu	38.88±15.72	40.50±14.72	55.83±11.72^###^	42.25±14.39***
HFnu	60.86±15.85	59.50±17.28	44.12±10.26^###^	57.62±14.43***
LF-HF ratio	0.61±0.27	0.67±0.32	1.36±0.48^###^	0.85±0.5***

**Table 3:** Comparison of time domain indices of heart rate variability parameters between control subjects and overt hypothyroid patients before and after L-thyroxine therapy Data presented are mean±SD. The P values less than 0.05 were considered statistically significant. *Indicates comparison between before six months and after six months in the control group and comparison between before six months and after six months in the overt hypothyroid group (*P<0.05, **P<0.01; ***P<0.001). ^#^Indicates comparison of before six months of the hypothyroid group with before six months of the control group (^#^P<0.05, ^##^P<0.01; ^###^P<0.001). RMSSD: square root of the mean of squares of the differences between adjacent NN intervals; SDNN: standard deviation of normal to normal interval; NN50: number of interval differences of successive NN intervals greater than 50; pNN50: proportion derived by dividing NN50 by the total number of NN intervals.

Parameters	Control group		Overt hypothyroid group	
	Before 6 months (n=42)	After 6 months (n=42)	Before 6 months (n=42)	After 6 months (n=42)
RMSSD (ms)	56.11±18.80	53.20±14.37	40.97±12.54^###^	45.65±16.37
SDNN	42.11±20.03	40.35±13.26	26.38±15.69^##^	41.54±36.31
NN50*	50.81±24.70	48.31±21.52	42.40±14.11	49.45±25.58
pNN50	26.37±10.35	25.82±10.42	20.5±4.56^#^	24.07±7.74

In the time domain indices, the standard deviation of normal to normal interval (SDNN) was significantly increased (P<0.01) after six months of L-thyroxine therapy in the overt hypothyroid group (Table 3).

Among the CAFT parameters, there was no significant difference between the pre-treatment and six-month post-treatment periods in the control group. In the overt hypothyroid group, the 30:15 ratio and ∆DBPIHG were significantly decreased (P<0.001) after six months of L-thyroxine therapy (Table 4).

**Table 4:** Comparison of cardiovascular autonomic function test parameters between control subjects and overt hypothyroid patients before and after L-thyroxine therapy Data presented are mean±SD. The P values less than 0.05 were considered statistically significant. *Indicates comparison between before six months and after six months in the control group and comparison between before six months and after six months in the overt hypothyroid group (*P<0.05, **P<0.01; ***P<0.001). ^#^Indicates comparison of before six months of the hypothyroid group with before six months of the control group (^#^P<0.05, ^##^P<0.01; ^###^P<0.001). ^†^Indicates comparison of after six months of the hypothyroid group with after six months of the control group (^†^P<0.05, ^††^P<0.01; ^†††^P<0.001). 30:15 ratio: ratio of maximum RR interval at 30th beat to minimum RR interval at 15th beat following standing; E:I ratio: ratio of average RR interval during expiration to that of during inspiration in six cycles of deep breathing; DDBPIHG: maximum rise in DBP above baseline following 30% of maximum voluntary contraction by isometric handgrip method.

Parameters	Control group		Overt hypothyroid group	
	Before 6 months (n=42)	After 6 months (n=42)	Before 6 months (n=42)	After 6 months (n=42)
30:15 ratio	1.26±0.11	1.28±0.12	1.46±0.08^###^	1.33±0.10***
E:I ratio	1.44±0.15	1.45±0.14	1.28±0.15^###^	1.27±0.12^†††^
∆DBP_IHG_	18.68±7.92	16.90±3.27	32.19±7.67^###^	17.52±5.08***

Biochemical profiles****

In the control group, biochemical parameters, like thyroid profile, lipid profile, and lipid risk factors, did not show any significant difference between the period before treatment and after six months of follow-up (Tables 5-8). In the overt hypothyroid group, six months follow-up after L-thyroxine therapy showed a significant increase in fT3 and fT4 (P<0.001) and a significant decrease in TSH (P<0.001) (Table 5).

**Table 5:** Comparison of thyroid profile between control subjects and overt hypothyroid patients before and after L-thyroxine therapy Data presented are mean±SD. The P values less than 0.05 were considered statistically significant. *Indicates comparison between before six months and after six months in the control group and comparison between before six months and after six months in the overt hypothyroid group (*P<0.05, **P<0.01; ***P<0.001). ^#^Indicates comparison of before six months of the hypothyroid group with before six months of the control group (^#^P<0.05, ^##^P<0.01; ^###^P<0.001). TSH: thyroid-stimulating hormone.

Parameters	Control group		Overt hypothyroid group	
	Before 6 months (n=42)	After 6 months (n=42)	Before 6 months (n=42)	After 6 months(n=42)
Free T_3_	2.91±0.41	2.78±0.37	1.57±0.59^###^	2.95±0.70***
Free T_4_	1.29±0.21	1.32±0.25	0.63±0.27^###^	1.50±0.60***
TSH	2.36±1.097	2.85±1.11	95.52±60.78^###^	6.55±10.87***

**Table 6:** Comparison of lipid profile between control subjects and overt hypothyroid patients before and after L-thyroxine therapy Data presented are mean±SD. The P values less than 0.05 were considered statistically significant. *Indicates comparison between before six months and after six months in the control group and comparison between before six months and after six months in the overt hypothyroid group (*P<0.05, **P<0.01; ***P<0.001). ^#^Indicates comparison of before six months of the hypothyroid group with before six months of the control group (^#^P<0.05, ^##^P<0.01; ^###^P<0.001). ^†^Indicates comparison of after six months of the hypothyroid group with after six months of the control group (^†^P<0.05, ^††^P<0.01; ^†††^P<0.001). TC: total cholesterol; TG: triglycerides; LDL-C: low-density lipoprotein cholesterol; VLDL-C: very-low-density lipoprotein cholesterol; HDL-C: high-density lipoprotein cholesterol; AI: atherogenic index = log (TG/HDL).

Parameters	Control group		Overt hypothyroid group	
	Before 6 months (n=42)	After 6 months (n=42)	Before 6 months (n=42)	After 6 months (n=42)
TC (mg/dL)	165.52±34.23	170.51±33.45	275.64±44.23^###^	189.83±27.80***
TG (mg/dL)	63.79±14.83	65.82±12.42	133.88±30.63^###^	95.21±31.38***,^†††^
HDL-C (mg/dL)	46.13±10.91	42.32±9.75	36.21±11.02^###^	42.50±5.62*
LDL-C (mg/dL)	106.62±32.41	110.72±31.25	212.65±46.83^###^	135.59±26.02***,^††^
VLDL-C (mg/dL)	12.75±2.96	13.45±3.27	26.77±6.12^###^	31.74±6.27***,^†††^

**Table 7:** Comparison of lipid risk factors between control subjects and overt hypothyroid patients before and after L-thyroxine therapy Data presented are mean±SD. The P values less than 0.05 were considered statistically significant. *Indicates comparison between before six months and after six months in the control group and comparison between before six months and after six months in the overt hypothyroid group (*P<0.05, **P<0.01; ***P<0.001). ^#^Indicates comparison of before six months of the hypothyroid group with before six months of the control group (^#^P<0.05, ^##^P<0.01; ^###^P<0.001). ^†^Indicates comparison of after six months of the hypothyroid group with after six months of the control group (^†^P<0.05, ^††^P<0.01; ^†††^P<0.001). TC: total cholesterol; TG: triglycerides; LDL-C: low-density lipoprotein cholesterol; HDL-C: high-density lipoprotein cholesterol; AI: atherogenic index = log10 (TG/HDL).

Parameters	Control group		Overt hypothyroid group	
	Before 6 months (n=42)	After 6 months (n=42)	Before 6 months (n=42)	After 6 months (n=42)
TC/HDL	3.72±0.97	4.05±0.75	7.62±3.05^###^	4.45±0.88***
TG/HDL	1.40±0.47	1.56±0.52	3.70±1.47^###^	2.23±0.76***,^††^
LDL/HDL	2.32±0.90	2.65±0.82	5.87±2.81^###^	3.19±0.83***
AI	0.15±0.10	0.19±0.13	0.56±0.15^###^	0.34±0.15***,^†††^

**Table 8:** Comparison of immunological, inflammatory, and oxidative stress markers between control subjects and overt hypothyroid patients before and after L-thyroxine therapy Data presented are mean±SD. The P values less than 0.05 were considered statistically significant. *Indicates comparison between before six months and after six months in the control group and comparison between before six months and after six months in the overt hypothyroid group (*P<0.05, **P<0.01; ***P<0.001). ^#^Indicates comparison of before six months of the hypothyroid group with before six months of the control group (^#^P<0.05, ^##^P<0.01; ^###^P<0.001). ^†^Indicates comparison of after six months of the hypothyroid group with after six months of the control group (^†^P<0.05, ^††^P<0.01; ^†††^P<0.001). Anti-TPO Ab: anti-thyroperoxidase antibody; Anti-Tg Ab: anti-thyroglobulin antibody; hsCRP: high-sensitivity C-reactive protein; IgA: immunoglobulin A; IgG: immunoglobulin G; IgE: immunoglobulin E; MDA: malondialdehyde.

Parameters		Control group		Overt hypothyroid group	
		Before 6 months (n=42)	After 6 months (n=42)	Before 6 months (n=42)	After 6 months (n=42)
Anti-TPO (IU/mL)		35.21±13.85	40.56±15.28	406.09±103.78^###^	275.92±188.64***^,†††^
Anti-Tg (IU/mL)		1.35±0.82	1.57±0.77	81.81±20.42^###^	32.99±69.36***^,†††^
IgA (mg/dL)		147.03±107.29	152.99±98.56	222.03±137.29^#^	163.99±100.61
IgG (mg/dL)		755.58±437.35	780.90±460.65	922.80±540.93	257.49±302.42***
IgE (IU/mL)		137.90±95.72	142.38±72.69	225.51±106.63	164.03±63.48
hsCRP (ng/mL)	477.96±209.15		525.84±198.70	1086.18±704.59^###^	839.61±165.34*^,††^
MDA (µM/L)	3.43±1.13		3.75±1.12	19.03±7.93^###^	13.73±5.98***^,†††^

Lipid profile parameters, like TC, TG, LDL-C, VLDL-C, and lipid risk factors, were significantly decreased (P< 0.001) after six months of L-thyroxine therapy in the overt hypothyroid group (Tables 6, 7). The HDL-C was significantly increased (P< 0.05) after six months of L-thyroxine therapy in the overt hypothyroid group (Table 6).

There was no significant difference in anti-TPO, anti-TG, hs-CRP, and MDA between before and after six months in the control group (Table 8). However, anti-TPO, anti-TG, MDA (P<0.001), and hs-CRP (P<0.05) were significantly decreased after six months of L-thyroxine therapy in the overt hypothyroid group (Table 8).

Regression analysis****

Multiple regression analysis demonstrated the significant association of the LF-HF ratio with MAP, AI, hs-CRP, and MDA, but not BMI, before L-thyroxine treatment in the overt hypothyroid group (Table 9). However, after L-thyroxine treatment, MDA was significantly associated with the LF:HF ratio in the overt hypothyroid group (Table 9).

**Table 9:** Multiple regression analysis of LF-HF ratio (as dependent variable) with various other associated factors (as independent variables) in overt hypothyroid patients before treatment and after six months of treatment (n=42). The P values less than 0.05 were considered statistically significant. CI: 95% confidence interval of unstandardized β; BMI: body mass index; MAP: mean arterial pressure; AI: atherogenic index; hsCRP: high-sensitivity C-reactive protein; MDA: malondialdehyde.

Independent variables	Before treatment		After treatment	
	Standardized regression coefficient B (95% CI)	P-value	Standardized regression coefficient B (95% CI)	P-value
BMI	0.075 (-0.082 to 0.095)	0.378	0.098 (-0.032 to 0.057)	0.312
MAP	0.326 (0.137 to 1.246)	0.012	0.145 (-0.004 to 0.035)	0.187
AI	0.568 (0.216 to 2.102)	0.003	0.235 (-0.003 to 0.027)	0.072
hsCRP	0.472 (-1.206 to 0.378)	0.008	0.204 (-0.022 to 0.000)	0.090
MDA	0.330 (-1.109 to 0.470)	0.011	0.271 (-1.354 to 0.628)	0.042

## Discussion

Forty-two overt hypothyroid patients were followed for six months of L-thyroxine therapy to assess the status of improvement in their autonomic functions and reduction in CV risks post-therapy. Follow-up compliance in the present study for post-treatment check-ups was 39.62% (42 out of 106 reported for follow-up). During their regular visits, general data regarding patient symptoms was collected, and after three months of treatment, many of them had reported a significant reduction in their symptoms like decreased tiredness and puffiness of the body, and substantial improvement in their conditions like appetite, muscular strength and bowel habits, which might be one of the reasons for the dropout rate of the study.

As expected, after six months of L-thyroxine treatment, T3 and T4 values were normalized in hypothyroid subjects (Table 5). TSH value was also considerably reduced after six months of treatment, further confirming the normalization of the thyroid profile. BMI and DBP values were also normalized following six months of therapy (Table 1). Thus, normalization of CV parameters and near-normalization of thyroid profile indicate substantial improvement in the condition of the patients, and six months of L-thyroxine therapy appears to be quite effective in the treatment of hypothyroidism. Similar reports were observed in SCH and OH patients after receiving L-thyroxine for long term [21]. Among the HRV indices, LFnu was reduced significantly, indicating a reduction in sympathetic drive, and HFnu was increased significantly, indicating improvement in parasympathetic drive following thyroxine treatment [22]. Change in LFnu and HFnu resulted in normalization of the LF-HF ratio (Table 2) in hypothyroid patients post-treatment, as these HRV indices following six months of treatment with thyroxine in hypothyroid patients were similar to the values of the control group, indicating the restoration of sympathovagal balance [14,22]. However, TP was not fully increased to the normal level, as there was a significant difference (P<0.05) in control after six months and hypothyroid after six months (Table 2). As TP is representative of HRV, and a reduction in TP per se is a CV risk [23], significantly less TP in hypothyroid patients following six months of treatment indicates that these subjects are still vulnerable to CV risks. Among time-domain indices, RMSSD is an important marker of vagal cardiac modulation. The persistence of significantly less RMSSD (Table 3) following six months of treatment with thyroxine in hypothyroid patients indicates that they still have less vagal activity [14]. Among the autonomic reactivity tests, though the 30:15 ratio and ∆DBPIHG were significantly (P<0.001) decreased after six months of L-thyroxine therapy, there was no significant change in the E:I ratio in response to deep breathing (Table 4). Thus, vagal reactivity continued to be depressed in hypothyroid patients after six months of eltroxin therapy.

In the overt hypothyroid patients, lipid parameters (TC, TG, LDL, and VLDL) were significantly decreased following six months of treatment in hypothyroid patients compared to their pre-treatment values, and the values were not fully normalized compared to the levels of lipids in the control group. In particular, TG, LDL, and VLDL were still significantly high in patients after six months compared to controls after six months (Table 6). Also, the lipid risk factors (TG/HDL, LDL/HDL, and AI) were still high in hypothyroid patients following six months of thyroxine treatment (Table 7). Increased lipid risk factors in these patients are potential causes of CV disease risk. Also, the level of MDA was significantly higher in hypothyroid patients following six months of thyroxine treatment, compared to control values (Table 8). The persistence of hyperlipidemia in hypothyroid patients could be the cause of oxidative stress (high MDA), which further predisposes them to CV risks. Although the immunological markers (anti-TPO Ab, anti-Tg Ab) and inflammatory marker (hs-CRP) were decreased substantially in the hypothyroid patients following six months of thyroxine therapy compared to their pre-treatment values, they were still significantly high compared to control values (Table 8), indicating that these patients continued to have low-grade immunological inflammation. The significant independent contribution of AI, hs-CRP, and MDA to LF-HF ratio of HRV as demonstrated by multiple regression analysis (Table 9) indicates that decreased HRV in hypothyroid patients following six months of thyroxine therapy is likely due to the persistence of metabolic dysfunctions in the form of dyslipidemia, oxidative stress, and retrograde inflammation. Among these factors, oxidative stress could be contributing maximally to the decreased HRV, as MDA level was considerably high in hypothyroid patients following six months of thyroxine therapy, and MDA had a significantly higher association with LF-HF ratio compared to AI and hsCRP (Table 9).

TSH plays a key role in maintaining SVI, as the sympathetic production is stimulated by TSH in the central nervous system. The findings from the present study indicate that the sympathetic reactivity was normalized in hypothyroid patients following six months of thyroxine therapy, which might be due to the near-normalization of TSH levels in hypothyroid patients after six months of eltroxin/L-thyroxine treatment. However, the parasympathetic reactivity was not substantially improved in them. Also, decreased HRV and RMSSD, decreased E:I ratio, increased lipid risk factors, and oxidative and inflammatory markers indicate that these patients still have cardiometabolic risks after six months of treatment, indicating partial reversibility of the autonomic damage in the following six months of eltroxin therapy. It might be that the autonomic nervous system had adapted to the irreversible changes due to its long-term exposure to hypothyroidism, as OH shows more severe complications than SCH. It is also acceptable that the duration of the treatment might be one of the reasons for the lack of complete reversibility of this damage, which would suggest that the autonomic imbalance might be restored at more than six months of L-thyroxine treatment. There were no adverse events that occurred after giving the L-thyroxine therapy.

Limitations

The present study has certain limitations that need to be acknowledged. We did not assess cardiac dysfunction using advanced radio-imaging techniques or echocardiography, which could have provided detailed insights into structural and functional cardiac changes. Additionally, 24-hour recordings of HRV, continuous BPV, and BRS were not performed, which might have offered a more comprehensive evaluation of cardiovascular dysfunction in overt hypothyroid patients. Furthermore, due to financial constraints, we were unable to estimate key cardiometabolic markers such as apolipoprotein A1, apolipoprotein B, lipoprotein(a), homocysteine, adiponectin, leptin, resistin, interleukin-6, and biochemical markers of sympathetic activity, including catecholamines and their metabolites in plasma or urine, which could have provided a more robust assessment of cardiovascular health and risk. Finally, the impact of lifestyle modifications, such as diet and exercise, was not studied in this follow-up due to time constraints, which might have influenced the cardiometabolic outcomes observed in the study.

## Conclusions

The present study demonstrates that six months of L-thyroxine therapy effectively normalizes thyroid hormone levels (TSH, fT3, fT4) and partially restores sympathovagal balance in OH patients, as indicated by improved LF-HF ratios and reduced sympathetic dominance. However, residual autonomic dysfunction, evidenced by persistently reduced TP, RMSSD, and vagal reactivity, along with ongoing dyslipidemia (elevated TG, LDL-C, VLDL-C), oxidative stress (high MDA), and chronic inflammation (raised hsCRP and anti-thyroid antibodies), suggests incomplete cardiometabolic recovery within the studied timeframe. These findings highlight the necessity for clinicians to monitor autonomic and metabolic parameters beyond biochemical euthyroidism, emphasizing that cardiovascular risks may persist despite standard thyroid hormone replacement therapy.

Future research should focus on extended longitudinal studies exceeding 12 months to determine the optimal duration required for complete autonomic and metabolic recovery in OH patients. Additionally, exploring adjunct therapeutic strategies, such as lipid-lowering agents, antioxidant supplementation, and anti-inflammatory interventions, could provide comprehensive cardiovascular risk reduction. Furthermore, investigations into lifestyle modifications, including dietary approaches and structured exercise programs, and the development of integrated biomarker panels (e.g., combining oxidative stress, inflammation, and lipid markers) could enhance early identification and targeted management of residual cardiometabolic risks in hypothyroid populations.

## References

[1] Unnikrishnan AG, Kalra S, Sahay RK, Bantwal G, John M, Tewari N (2013). Prevalence of hypothyroidism in adults: An epidemiological study in eight cities of India. Indian J Endocrinol Metab.

[2] Garber JR, Cobin RH, Gharib H (2012). Clinical practice guidelines for hypothyroidism in adults: Cosponsored by the American Association of Clinical Endocrinologists and the American Thyroid Association. Endocr Pract.

[3] Bricker LA, Such F, Loehrke ME, Kavanaugh K (2001). Intractable diarrhea in hyperthyroidism: Management with beta-adrenergic blockade. Endocr Pract.

[4] Burggraaf J, Tulen JH, Lalezari S (2001). Sympathovagal imbalance in hyperthyroidism. Am J Physiol Endocrinol Metab.

[5] Galetta F, Franzoni F, Fallahi P, Tocchini L, Braccini L, Santoro G, Antonelli A (2008). Changes in heart rate variability and QT dispersion in patients with overt hypothyroidism. Eur J Endocrinol.

[6] Karthik S, Pal GK, Nanda N, Hamide A, Bobby Z, Amudharaj D, Pal P (2009). Sympathovagal imbalance in thyroid dysfunctions in females: Correlation with thyroid profile, heart rate and blood pressure. Indian J Physiol Pharmacol.

[7] Syamsunder AN, Pal P, Kamalanathan CS (2014). Dyslipidemia and low-grade inflammation are associated with sympathovagal imbalance and cardiovascular risks in subclinical and overt hypothyroidism. Int J Clin Exp Physiol.

[8] Syamsunder AN, Pal P, Pal GK, Kamalanathan CS, Parija SC, Nanda N, Sirisha A (2017). Decreased baroreflex sensitivity is linked to the atherogenic index, retrograde inflammation, and oxidative stress in subclinical hypothyroidism. Endocr Res.

[9] Syamsunder AN, Pal GK, Pal P, Kamalanathan CS, Parija SC, Nanda N (2013). Association of sympathovagal imbalance with cardiovascular risks in overt hypothyroidism. N Am J Med Sci.

[10] Moon S, Kim MJ, Yu JM, Yoo HJ, Park YJ (2018). Subclinical hypothyroidism and the risk of cardiovascular disease and all-cause mortality: A meta-analysis of prospective cohort studies. Thyroid.

[11] Rousset B, Dupuy C, Miot F, Dumont J (2000). Chapter 2 Thyroid hormone synthesis and secretion. Endotext [Internet].

[12] Fish LH, Schwartz HL, Cavanaugh J, Steffes MW, Bantle JP, Oppenheimer JH (1987). Replacement dose, metabolism, and bioavailability of levothyroxine in the treatment of hypothyroidism. Role of triiodothyronine in pituitary feedback in humans. N Engl J Med.

[13] Chen Y, Tai HY (2020). Levothyroxine in the treatment of overt or subclinical hypothyroidism: A systematic review and meta-analysis. Endocr J.

[14] (1996). Heart rate variability: standards of measurement, physiological interpretation and clinical use. Task Force of the European Society of Cardiology and the North American Society of Pacing and Electrophysiology. Circulation.

[15] Low PA (2004). Laboratory evaluation of autonomic function. Suppl Clin Neurophysiol.

[16] Weimer LH (2010). Autonomic testing: Common techniques and clinical applications. Neurologist.

[17] Rovere MT, Maestri R, Pinna GD (2011). Baroreflex sensitivity assessment - latest advances and strategies. Eur Cardiol.

[18] Imholz BP, Wieling W, van Montfrans GA, Wesseling KH (1998). Fifteen years experience with finger arterial pressure monitoring: Assessment of the technology. Cardiovasc Res.

[19] Dobiásová M (2004). Atherogenic index of plasma [log(triglycerides/HDL-cholesterol)]: Theoretical and practical implications. Clin Chem.

[20] Khandelwal D, Tandon N (2012). Overt and subclinical hypothyroidism: Who to treat and how. Drugs.

[21] Razvi S, Ingoe L, Keeka G, Oates C, McMillan C, Weaver JU (2007). The beneficial effect of L-thyroxine on cardiovascular risk factors, endothelial function, and quality of life in subclinical hypothyroidism: Randomized, crossover trial. J Clin Endocrinol Metab.

[22] Malliani A (2005). Heart rate variability: From bench to bedside. Eur J Intern Med.

[23] Tsuji H, Larson MG, Venditti FJ Jr, Manders ES, Evans JC, Feldman CL, Levy D (1996). Impact of reduced heart rate variability on risk for cardiac events. The Framingham Heart Study. Circulation.

